# Phytic Acid Decreases Oxidative Stress and Intestinal Lesions Induced by Fumonisin B_1_ and Deoxynivalenol in Intestinal Explants of Pigs

**DOI:** 10.3390/toxins11010018

**Published:** 2019-01-04

**Authors:** Elisângela O. da Silva, Juliana R. Gerez, Miriam S. N. Hohmann, Waldiceu A. Verri, Ana Paula F. R. L. Bracarense

**Affiliations:** 1 Laboratório de Anatomia Patológica Veterinária, Universidade do Oeste Paulista, Presidente Prudente, São Paulo 19050-920, Brazil; elivet02@gmail.com; 2 Laboratory of Animal Pathology, Universidade Estadual de Londrina, Londrina, Paraná 86057-970, Brazil; julianarubira@hotmail.com; 3 Laboratory of Pain, Inflammation, Neuropathy and Cancer, Universidade Estadual de Londrina, Londrina, Paraná 86057-970, Brazil; miriam_snh15@hotmail.com (M.S.N.H.); waldiceujr@yahoo.com.br (W.A.V.J.)

**Keywords:** mycotoxins, IP6, reactive oxygen species, morphology, jejunum, pigs

## Abstract

The purpose of the present study was to investigate the effects of phytic acid (IP6) on morphological and immunohistochemical parameters and oxidative stress response in intestinal explants of pigs exposed to fumonisin B_1_ (FB_1_) and/or deoxynivalenol (DON). The jejunal explants were exposed to the following treatments: vehicle, IP6 5 mM, DON 10 µM, FB_1_ 70 µM, DON 10 µM + FB_1_ 70 µM, DON 10 µM + IP6 5 mM, FB_1_ 70 µM + IP6 5 mM, and DON 10 µM + FB_1_ 70 µM + IP6 5 mM. The decrease in villus height and goblet cell density was more evident in DON and DON + FB_1_ treatments. In addition, a significant increase in cell apoptosis and cell proliferation and a decrease in E-cadherin expression were observed in the same groups. DON and FB_1_ exposure increased cyclooxygenase-2 expression and decreased the cellular antioxidant capacity. An increase in lipid peroxidation was observed in DON- and FB_1_-treated groups. IP6 showed beneficial effects, such as a reduction in intestinal morphological changes, cell apoptosis, cell proliferation, and cyclooxygenase-2 expression, and an increase in E-cadherin expression when compared with DON, FB_1_ alone, or DON and FB_1_ in association. IP6 inhibited oxidative stress and increased the antioxidant capacity in the explants exposed to mycotoxins.

## 1. Introduction

Fumonisin B_1_ (FB_1_) and deoxynivalenol (DON) are the most frequently occurring mycotoxin contaminants in agricultural commodities worldwide, and represent a risk for human and animal health [1]. Exposure to mycotoxins is inevitable, therefore effective strategies to mitigate or even eliminate their harmful impacts are required. In recent years, research involving nutraceutical substances and compounds has increased, due to the excellent preventive and therapeutic action of these compounds on the mycotoxins’ toxic effects [2].

FB_1_ and DON are mycotoxins, mainly produced by *Fusarium* spp., that commonly contaminate maize, wheat, barley, and oats [3]. The intracellular action of these mycotoxins has been elucidated, and the induction of oxidative stress and generation of radical oxygen species (ROS) play an important role in their toxic effects, as observed in vivo [4,5] and in vitro [6,7]. Other toxic effects, such as altered intestinal morphology (expression of cell junction proteins, cell proliferation and apoptosis, production of mucin) and inflammatory response (deregulation of anti and pro-inflammatory cytokines and overexpression of cyclooxygenase-2 (cox-2), have also been reported [8,9,10,11,12]. However, the association between intestinal lesions and oxidative stress induced by FB_1_ and DON has not been elucidated.

Phytic acid (IP6) is a natural antioxidant widely present in cereals and legumes [13]. Several studies have demonstrated the preventive and therapeutic effects of IP6 in diseases associated with mineral and endocrine disturbances, chronic inflammation, and cancer development [14,15,16]. Specifically in models of intestinal inflammation and cancer, IP6 has shown protective effects by decreasing aberrant crypt formation, increasing cell viability, and downregulating pro-inflammatory cytokine, chemokine and cox-2 expression [17,18,19].

Swine are one of the most sensitive species to the toxic effects of FB_1_ and DON [9]. Furthermore, the similarities with the human immune system and intestinal physiology, as well as with the absorption of IP6 [20], make pigs the ideal experimental model to study the toxic effects induced by mycotoxins, and strategies to mitigate the toxicity using IP6. However, most studies focusing on the effects of FB_1_ and DON on oxidative stress have been performed in vitro or using laboratory mammals and chickens [21].

In previous in vitro [22] and ex vivo [12] studies, we have shown that IP6 exposure decreases the intestinal lesions induced by FB_1_ and DON. However, the effect of IP6 on oxidative stress response produced by mycotoxins in the intestine remains to be determined, as well as the effect of IP6 on the combined effects of FB_1_ and DON. Therefore, the aim of the present study was to investigate the action of IP6 on the toxic effects induced by FB_1_ and DON alone, or in FB_1_ and DON in association on the intestine, focusing on the oxidative stress response, morphology, goblet cell density, cell proliferation and apoptosis, and E-cadherin and cox-2 expression in jejunal explants of pigs.

## 2. Results

### 2.1. Morphological Assessment

**Figure 1:**
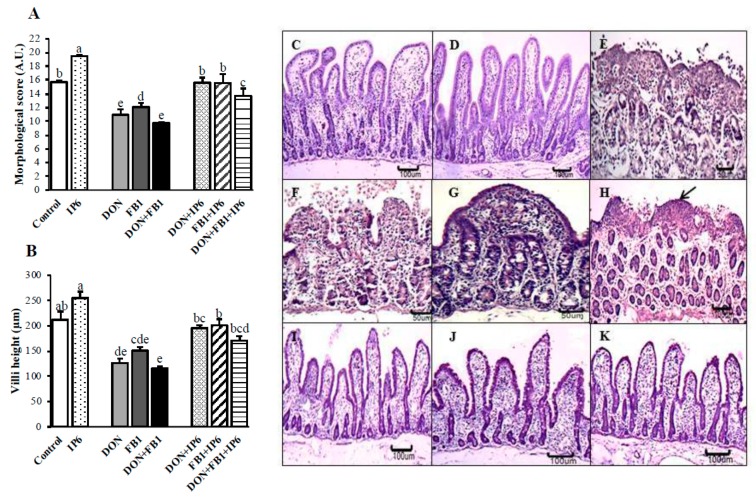
Effects of deoxynivalenol (DON) and fumonisin FB_1_ (FB_1_), alone or in association, and phytic acid (IP6) on histological morphology in jejunal explants. (**A**) Morphological score (AU—Arbitrary Units). (**B**) Villi height (µm). Explants exposed to control treatment (□); IP6 5 mM (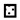); DON (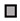); FB_1_ (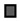); DON + FB_1_ (■); DON + IP6 (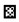); FB_1_ + IP6 (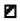); DON + FB_1_ + IP6 (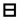). Mean values with different superscript letters were significantly different (*p* ≤ 0.05). (**C**) Control treatment. Hematoxylin-eosin (HE), bar 100 µm. (**D**) IP6 5 mM treatment. HE, bar 100 µm. (**E**) DON 10 µM alone: severe villi atrophy and fusion, and loss of the apical enterocytes. HE, bar 50 µm. (**F**) FB_1_ alone: severe loss of apical enterocytes. HE, bar 50 µm. (**G**) DON 10 µM + FB_1_: severe villi fusion. HE, bar 50 µm. (**H**) DON 10 µM + FB_1_: flattened enterocytes (arrow), villi atrophy and loss of enterocytes. HE, bar 100 µm. (**I**) DON 10 µM + IP6 5 mM: histological aspects similar to the control group. HE, bar 100 µm. (**J**) FB_1_ + IP6 5 mM: histological aspects similar to the control group. HE, bar 100 µm. (**K**) DON 10 µM plus FB_1_ + IP6 5 mM: histological aspects similar to the control group. HE, bar 100 µm.

After 3 h of exposure to DON and/or FB_1_, the explants exhibited moderate to severe jejunal lesions, mainly in the DON + FB_1_ treatment group (Figure 1A). The main histological changes observed included multifocal to diffuse cytoplasmic vacuolation and flattening of enterocytes, atrophy and villi fusion, lack of apical epithelium, and necrotic debris (Figure 1E–G). Morphological scores decreased significantly in the explants exposed to DON (30.2%), FB_1_ (23%), and DON + FB_1_ (38%) compared with the control group. The jejunum exposed to DON presented a significant decrease of 9.4% in the histological score compared to FB_1_ alone (*p* ≤ 0.05), and the treatment DON + FB_1_ showed a significant decrease of 23.3% compared to FB_1_ (*p* ≤ 0.05).

Alternatively, when explants were pre-treated with IP6, the histological scores exhibited a significant increase when compared with explants exposed to DON or FB_1_ alone, or DON and FB_1_ in association (Figure 1A). Explants subjected to IP6 alone presented a significant improvement (*p* ≤ 0.05) of 24.2% in the morphological score when compared to the control (Figure 1A,C,D). In addition, explants exposed to IP6 plus mycotoxins exhibited increases of 42.7% (DON + IP6), 28.6% (FB_1_ + IP6), and 40.4% (DON + FB_1_ + IP6) compared to the respective treatments with the mycotoxins alone (*p* ≤ 0.05). The main histological lesions observed in the explants subjected to IP6 pre-treatment were cytoplasmic vacuolation and interstitial edema. The jejunal explants subjected to DON + IP6 or FB_1_ + IP6 showed lesional scores similar to the control treatment (*p* ≤ 0.05) (Figure 1A).

The jejunal explants exposed to mycotoxins exhibited a significant decrease (*p* ≤ 0.05) in villi height compared with the control (DON—40.4%, FB_1_—30%, and DON + FB_1_—45.4%). However, explants exposed to mycotoxins and treated with IP6 exhibited a significant increase (*p* ≤ 0.05) of 54.6% compared to DON alone, 33% compared to FB_1_ alone, and 47% compared to DON + FB_1_ (Figure 1B). The jejunal explants exposed to mycotoxins plus IP6 showed a villi height similar to the control explants (*p* ≤ 0.05) (Figure 1B,I–K).

**Figure 2:**
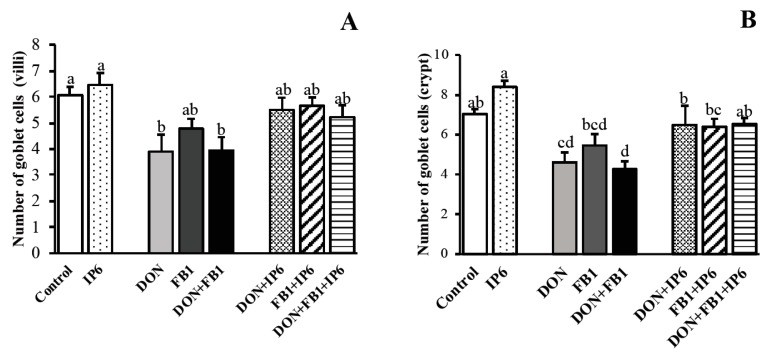
Mean goblet cell density on jejunal explants. (**A**) Number of goblet cells on the villi of jejunal explants. (**B**) Number of goblet cells on the crypts of jejunal explants. Explants exposed to control treatment (□); IP6 5 mM (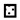); deoxynivalenol (DON) (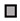); fumonisin B_1_ (FB_1_) (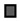); DON + FB_1_ (■); DON + IP6 (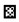); FB_1_ + IP6 (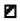); DON + FB_1_ + IP6 (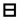). Mean values with different superscript letters were significantly different (*p* ≤ 0.05).

The number of goblet cells in the villi of explants exposed to DON alone and DON + FB_1_ decreased significantly (*p* ≤ 0.05) when compared to the control (35.6% and 35%, respectively) (Figure 2). In the crypt region, the number of goblet cells decreased in DON (34.4%) and DON + FB_1_ (39%) treatments (*p* ≤ 0.05). Explants exposed to DON + IP6 and DON + FB_1_ + IP6 showed a significant increase (*p* ≤ 0.05) in goblet cell density in the crypt region, compared to DON alone (40.7%) and DON + FB_1_ (53%).

The morphological evaluation showed that the pre-treatment with IP6 in the jejunal explants exposed to mycotoxins resulted in an improvement in the histological score, villi height and goblet cell density. These explants remained histologically similar to the control group (*p* > 0.05) (Figure 1 and Figure 2).

### 2.2. Caspase-3, Ki-67, E-cadherin and cox-2 Expression

**Figure 3:**
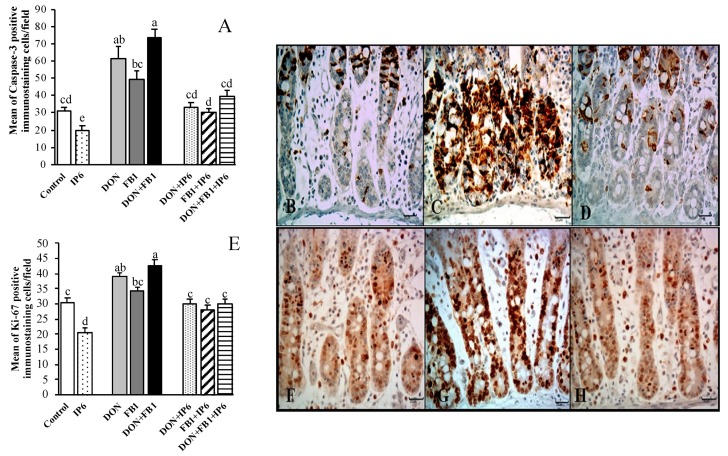
Effects of DON and FB_1_, alone or in association, and IP6 on apoptosis (Ccasp-3) and cell proliferation (Ki-67) in jejunal explants. Explants exposed to control treatment (□); IP6 5 mM (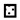); deoxynivalenol (DON) (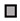); fumonisin B_1_ (FB_1_) (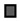); DON + FB_1_ (■); DON + IP6 (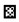); FB_1_ + IP6 (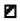); DON + FB_1_ + IP6 (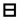). (**A**) Mean number of Ccasp-3 immunostained cells per field on explants exposed to different treatments. Mean values with different superscript letters were significantly different (*p* ≤ 0.05). (**B**) Control treatment: mild Ccasp-3 cytoplasmic immunostaining in crypt cells. (**C**) DON 10 µM + FB_1_ 70 µM: diffuse Ccasp-3 cytoplasmic immunostaining in crypt cells. (**D**) DON 10 µM + FB_1_ 70 µM + IP6 5 mM: decrease in Ccasp-3 cytoplasmic immunostaining in crypt cells. (**E**) Mean number of Ki-67 immunostained cells per field on explants exposed to different treatments. Mean values with different superscript letters were significantly different (*p* ≤ 0.05). (**F**) Control treatment: mild Ki-67 nuclear immunostaining in crypt cells. (**G**) DON 10 µM + FB_1_ 70 µM: diffuse Ki-67 nuclear immunostaining in crypt cells. (**H**) DON 10 µM + FB_1_ 70 µM + IP6 5 mM: decrease in Ki-67 nuclear immunostaining in crypt cells. (**B**–**D**; **F**–**H**: immunoperoxidase method, bar 25 µm).

The expression of caspase-3 (Ccasp-3), an indicator of cell apoptosis, decreased 37.3% in the jejunal explants exposed to IP6 compared to the control treatment (*p* ≤ 0.05). A significant increase in Ccasp-3 expression was observed in explants exposed to DON (95.5%) and DON + FB_1_ (135.5%) when compared to the control group (*p* ≤ 0.05). In addition, an increase of 49.6% in cell apoptosis (*p* ≤ 0.05) was verified in the DON + FB_1_ group compared to FB_1_ alone. Nonetheless, a significant decrease in cell apoptosis was observed in DON + IP6 (46.8%), FB_1_ + IP6 (39%) and DON/FB_1_ + IP6 (46.8%) groups compared to the respective treatments with the mycotoxins alone (*p* ≤ 0.05) (Figure 3A–D).

Explants exposed to IP6 exhibited a decrease of 32.5% (*p* ≤ 0.05) in Ki-67 expression compared to the control samples. Meanwhile, an increase in cell proliferation (*p* ≤ 0.05) was observed in explants exposed to DON (28.4%) and DON + FB_1_ (40%) when compared to the control explants. Jejunal explants subjected to DON + FB_1_ treatment showed a significant increase (*p* ≤ 0.05) of 24.7% in cell proliferation compared to FB_1_ alone. The presence of IP6 induced a significant decrease (*p* ≤ 0.05) in the cell proliferation of the jejunal explants exposed to DON (30.3%) and DON + FB_1_ (29.6%), compared to explants exposed only to DON and DON + FB_1_ (Figure 3E–H).

**Figure 4:**
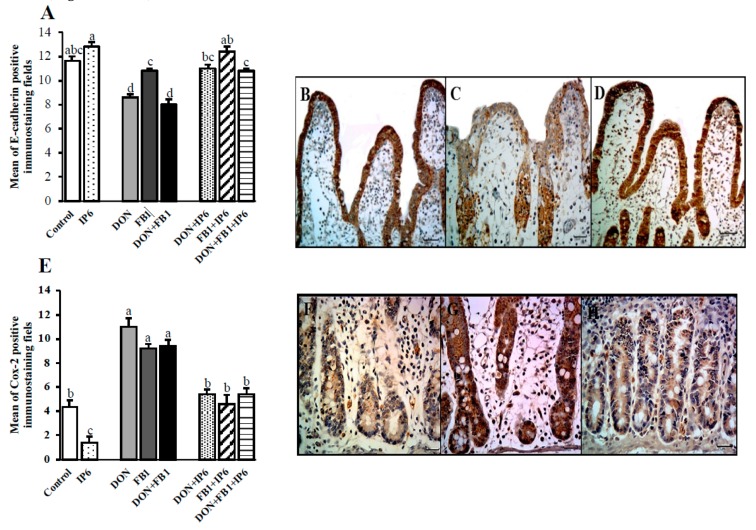
Effects of DON and FB_1_, alone or in association, and IP6 on E-cadherin and cox-2 expression in jejunal explants. Explants exposed to control treatment (□); IP6 5 mM (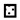); deoxynivalenol (DON) (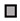); fumonisin B_1_ (FB_1_) (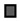); DON + FB1 (■); DON + IP6 (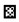); FB_1_ + IP6 (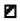); DON + FB_1_ + IP6 (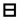). (**A**) Mean of E-cadherin positive immunostaining per fields on explants exposed to different treatments. Mean values with different superscript letters were significantly different (*p* ≤ 0.05). (**B**) Control treatment: strong and homogeneous E-cadherin immunostaining in epithelial cells. Bar 50 µm. (**C**) DON 10 µM: mild and non-homogeneous E-cadherin immunostaining in epithelial cells. Bar 25 µm. (**D**) DON 10 µM + IP6 5 mM: strong and homogeneous E-cadherin immunostaining in epithelial cells similar to control treatment. Bar 50 µm. (**B**–**D**: immunoperoxidase method). (**E**) Mean of cox-2 positive immunostaining per fields on explants exposed to different treatments. Mean values with different superscript letters were significantly different (*p* ≤ 0.05). (**F**) Control treatment: mild cox-2 cytoplasmic immunostaining in crypt cells. (**G**) DON 10 µM: diffuse and strong cox-2 cytoplasmic immunostaining in crypt cells. (**H**) DON 10 µM + IP6 5 mM: decrease in cox-2 cytoplasmic immunostaining in crypt cells similar to control treatment. (**F**–**H**: immunoperoxidase method, bar 25 µm).

In general, exposure to DON, alone or in association with FB_1_, induced a significant reduction in E-cadherin expression (25.8% and 31%, respectively compared to the control; 20.3% and 26%, respectively compared to the FB_1_ treatment). However, when explants were pre-incubated with IP6 and subsequently exposed to the mycotoxins, an increase in E-cadherin immunostaining was observed compared to explants exposed to DON (28%), FB_1_ (15%), and DON + FB_1_ (35%) (*p* ≤ 0.05) alone (Figure 4A–D).

The immunoexpression of cox-2 (parameter used to evaluate the inflammatory response) showed a decrease of 68% in the jejunum treated with IP6, compared with the control samples. In spite of this, explants subjected to the mycotoxins showed a significant increase in cox-2 expression (*p* ≤ 0.05) (150% for DON, 109% for FB_1_, and 113% for DON + FB_1_ compared to the control). The presence of IP6 induced a significant decrease (*p* ≤ 0.05) of 51%, 50%, and 45.2% in explants exposed to DON, FB_1_, and DON + FB_1_, respectively (Figure 4E–H).

Overall, explants exposed to mycotoxins and treated with IP6 presented Ccasp-3, Ki-67, E-cadherin, and cox-2 expression similar to the control explants, indicating a beneficial effect of this product in reducing the toxic effects of mycotoxins.

### 2.3. Oxidative Stress Evaluation

Jejunal exposure to DON and FB_1_ induced a significant increase in thiobarbituric acid reactive substance (TBARS) levels (62.8% and 54.2%, respectively), while exposure to IP6 resulted in decreased levels when compared to the control explants (40%). When the explants were exposed to IP6 prior to mycotoxins (DON, FB_1_, and DON+ FB_1_), the TBARS levels remained similar to those of control explants (Table 1).

The capacity to respond to oxidative stress was evaluated by determining the levels of reduced glutathione (GSH) and ferric-reducing antioxidant power (FRAP) and 2,2′-azino-bis(3-ethylbenzothiazoline-6-sulphonic acid (ABTS) assays. The jejunal explants exposed to mycotoxins showed a significant decrease in endogenous GSH levels compared to the control (DON 62%; FB_1_ 51.8%; DON + FB_1_ 46.4%) (Table 1). The presence of IP6 induced an increase in GSH levels of 131% and 105.5% compared to DON and FB_1_ alone; in the explants exposed to DON + FB_1_, IP6 increased the GSH content by 65.1%.

Explants exposed to mycotoxins presented a significant decrease in ABTS scavenging ability (DON 33.6%; FB_1_ 35.5%; DON + FB_1_ 31.1%) compared to the control explants, while pre-exposure to IP6 promoted a significant increase in ABTS scavenging ability in explants exposed to DON (67.9%), FB_1_ (107%), and DON + FB_1_ (105.4%) (Table 1). The ferric reducing ability was significantly increased in explants exposed to IP6 + FB_1_ (20.6%) and IP6 + DON + FB_1_ (21.4%), compared to explants exposed to these same mycotoxins (Table 1). Collectively, these data indicate that pre-treatment with IP6 inhibits mycotoxin-induced lipid peroxidation and reduction in cellular antioxidant capacity in jejunal explants.

## 3. Discussion

This study investigated the protective effect of IP6 on jejunal explants of pigs exposed to FB_1_ and DON. Intestinal morphological changes induced by these mycotoxins alone [12] or in combination [9] have been previously established, and were similar to those observed in the present study. The intestinal epithelial cell (IEC) changes, such as cytoplasmic vacuolation, flattening, necrosis, and loss of microvilli observed following exposure to mycotoxins were probably induced by an increase in cytoplasmic and mitochondrial permeability, which may be associated with ROS generation and resultant lipid peroxidation, as observed in vitro [6,7] and in vivo [4,5].

The reductions in the number of goblet cells and villi height, evidenced in DON and FB_1_ + DON groups, are probably linked to increased levels of apoptosis. This association is supported by increased caspase-3 immunostaining in these groups. DON induces apoptosis by direct lesion to the mitochondria, or by the extrinsic pathway of apoptosis through increased TNF-α as a consequence of intestinal inflammation [6]. Considering the concentration of DON used and the short period of incubation, the elevated apoptotic index observed may be associated with the activation of the intrinsic apoptosis pathway, i.e., oxidative stress and changes in mitochondrial membrane potential (MMP), deregulation of Bcl-2/Bax expression, release of cytochrome C, and consequent activation of caspase-3. In addition, it has been established that DON can modulate cell proliferation and apoptosis through activation of mitogen-activated protein kinases (MAPKs), mainly ERK1/2, JNK ½, and p38 [23,24,25,26]. This modulation in cell proliferation was observed in the present study. The explants exposed to DON alone and FB_1_ + DON showed discrete elevation of cell proliferation in the crypt region, which likely occurred as a tissue response to the IEC lesions and high apoptosis level induced by DON, and is evidenced by the increase in Ki-67 expression.

Alterations in cell junction proteins [10], trans-epithelial electrical resistance (TEER), and paracellular permeability [8] were associated with DON intestinal toxicity. In the present study, explants exposed to DON and FB_1_, alone or in combination, showed decreased E-cadherin expression compared to the control treatment. This reduction is possibly associated with oxidative stress and the morphological changes observed in the IEC, since DON-induced ROS inhibits protein synthesis [27] and MAPKs activation [8].

Besides deregulation of cell proliferation and apoptosis, oxidative stress can induce an overexpression of cox-2 [28]. Cox-2 is an enzyme involved in the metabolism of arachidonic acid, which is strongly induced by p38 MAPK activation and proinflammatory stimuli such as TNF-α, IL-1, IL-6, and IL-8 [28]. In previous studies in pigs, an upregulation in inflammatory cytokines [9,29] and an increase in the expression of cox-2 [12] was associated with DON and FB_1_ exposure. In the present study, the expression of cox-2 increased significantly in all mycotoxin treatments (DON, FB_1_, and DON + FB_1_) demonstrating its role as a biomarker of intestinal oxidative stress and inflammatory response in this model. Cox-2 itself can produce superoxide anions as a by-product of prostanoid synthesis [30]. Analysis for oxidative stress involves free radical generation (resulting in lipid peroxidation) and depletion of antioxidant compounds. In this study, mycotoxins, alone or in combination, affected both mechanisms. An increase in lipid peroxidation was observed mainly in the single agent treatments (~1.5-fold). On the other hand, the GSH, ABTS, and FRAP levels decreased after mycotoxin exposure (~2.2-, 1.5-, and 1.1-fold, respectively).

Reductions in superoxide dismutase (SOD), catalase (CAT), glutathione peroxidase (GPx), and GSH levels have been reported in in vitro and in vivo studies after DON [29,31,32] and FB_1_ exposure [5,7]. DON toxicity is associated with ROS generation and secondary changes to the lysossomal membrane, and increase in mitochondrial membrane permeability with consequent deregulation of Bcl-2/Bax genes, release of cytochrome C, and activation of caspase-3 [6,33]. Similar to DON, oxidative stress induced by FB_1_ results in mitochondrial lesion and activation of caspase-3 [6,34,35]. In general, the effects of DON on intestinal epithelial cells were more evident than those of FB_1_, however, no significant difference was verified in the oxidative stress response. We hypothesize that explants exposed to FB_1_ present an oxidative stress response similar to DON. However, we also consider that the high cell apoptosis index in explants exposed to DON left less cells to suffer oxidative stress. Therefore, the real potential of oxidative stress induced by DON was masked, since several studies have demonstrated that DON rapidly induces ribotoxic stress and ROS generation, mainly affecting cells with a high cell division index, such as intestinal epithelial cells [36]. The oxidative stress induced by FB_1_ occurs indirectly via the intracellular accumulation of sphingolipids [29], a toxic mechanism with slower progression compared to DON’s toxic effect at the ribosomal level.

The presence of IP6 induced a protective effect on all histological parameters evaluated. This effect could be observed by the increased histological score, villi height, and goblet cell density compared to the jejunal explants exposed to the mycotoxins alone and associated treatments. The beneficial effects of IP6 are associated with its antioxidant capacity, mainly its ability to inhibit the Fenton reaction and formation of hydroxyl radicals [37]. The evaluation of cell proliferation, apoptosis, and E-cadherin expression suggests that IP6 modulates the toxic effects by decreasing ROS generation and, consequently, cell permeability and MAPKs activation, which results in the maintenance of protein synthesis. The reduction in mycotoxin-induced apoptosis by IP6 demonstrates its potent antioxidant effect, since ROS generation is directly linked to apoptosis activation. Similar modulation of cell viability has been observed in inflammatory bowel studies [19,38,39]. IP6 induced a significant reduction in cox-2 expression compared to the explants exposed to all mycotoxin treatments. The reduction in cox-2 expression observed in the present study can be associated with the ability of IP6 to inhibit ROS production, lipid peroxidation, and inflammatory stimuli [40] induced by DON and FB_1_. Moreover, studies have demonstrated that IP6 reduces the expression of cox-2 by inhibition of p38 MAPK, as well as the conversion of arachidonic acid into prostaglandins and suppression of β-catenin activity [19,40,41].

By the evaluation of oxidative stress, the presence of IP6 decreased the levels of TBARS in the explants subjected to DON and FB_1_ treatments by 66.6% and 48.8%, respectively. The presence of IP6 reduced TBARS level by 48.8% compared with the combined mycotoxin treatment. Furthermore, IP6 promoted a significant increase in GSH level and antioxidant capacity in the jejunal explants exposed to mycotoxins. In agreement, the ability of IP6 to protect cells against oxidative stress has been associated with the inhibition of ROS generation, increase of GSH level, CAT, GPx, and SOD content, and decrease of lipid peroxidation (MDA) in hepatocarcinogenesis studies in rats [42,43,44]. Nevertheless, data on the effects of IP6 on intestinal oxidative stress are still scarce. The present results demonstrate that the protective effect of IP6 on the intestinal oxidative stress is associated with its capacity to mitigate lipid peroxidation and increase the antioxidant capacity of the tissue.

The morphological changes and oxidative stress response were more evident in explants exposed to DON and DON + FB_1_ treatments, and were associated with the rapid and direct induction of oxidative stress by DON- compared to FB_1_-induced injury. Although FB_1_ and DON co-contamination of food and feed has been reported worldwide, the effects observed in mycotoxin multi-contaminations depend on the concentration, period of exposition, experimental model, and species susceptibility [45].

## 4. Conclusions

The mycotoxins DON and FB_1_, alone or in combination, induced changes in the morphology, cell proliferation, apoptosis, and expression of proteins associated with cell junctions and inflammation of the intestine. These toxic effects are associated with an oxidative stress response, including changes in lipid peroxidation and antioxidant capacity. However, more studies are necessary to elucidate the mechanisms and intracellular signaling pathways that trigger oxidative stress induced by these mycotoxins at the intestinal level. Phytic acid exerts beneficial effects upon the jejunum, modulating the changes induced by the mycotoxins and protecting cells against oxidative stress. In this context, IP6 antioxidant additives may represent an efficient approach in mitigating and preventing the intestinal toxic effects of DON and FB_1_.

## 5. Materials and Methods

### 5.1. Animals and Reagents (FB_1_, DON, and Phytic Acid)

Five 24-day-old crossbred (Landrace × Large White × Duroc) piglets (7.9 kg ± 0.72) were used in the present study. The purified DON (Molecular weight (MW): 296.32; Sigma–Aldrich, St. Louis, MO, USA) and FB_1_ (MW: 721.83; Cayman Chemical Company, Ann Arbor, MI, USA) were dissolved in ultrapure water at final concentrations of 10 μM for DON and 70 μM for FB_1_, and stored at 4 °C. The concentrations of FB_1_ (70 µM) and DON (10 µM) used are equivalent to 50.5 and 3 mg/kg of feed, respectively.

The phytic acid salt (MW: 819; Sigma–Aldrich, St. Louis, MO, USA) was dissolved in distilled water to the concentration of 15 mM, and the pH adjusted to 7.2. The solution was stored at −20 °C before dilution in the explant culture media. The IP6 (5 mM), DON (10 μM), and FB_1_ (70 μM) concentrations used were chosen according to previous studies [10,12,23].

### 5.2. Ex Vivo Experimental Model

The experimental procedures on animals were approved by the ethics commission (CEUA/UEL/Brazil-process n° 4173.2014.05, Date of approval: 09 March 2014). The piglets were euthanized (acepromazine 1%, sodium pentobarbital 40 mg/Kg and KCl 15%) and fragments of the jejunum (5 cm) were sampled, washed with a buffer solution, and opened longitudinally. For each treatment, six explants were sampled using an 8 mm punch, resulting in 48 explants per piglet. The explants were incubated in 6-well plates (three explants/well) at 37 °C in a chamber under CO_2_-controlled conditions with orbital shaking. The following treatments were applied: culture media [DMEM, (Gibco-BRL Life Technologies, Carlsbad, CA, USA) plus penicillin/streptomycin (1.25 µL/mL, Gibco-BRL Life Technologies, Carlsbad, CA, USA), gentamicin (10 µL/mL, Novafarma, São Paulo, SP, Brazil), fetal bovine serum (100 µL/mL, Invitrogen, São Paulo, SP, Brazil), and l-glutamine (0.4 µL/mL, Sigma Aldrich, St. Louis, MO, USA)] (A, B, C and D treatments), or culture media with IP6 5 mM (E, F, G, and H treatments). The mycotoxins were added to the wells after one hour: DON (10 µM) in the B, D, F, and H treatments, and FB_1_ (70 µM) in the C, D, G, and H treatments. After a total period of incubation of four hours, the explants (three from each treatment) were fixed in 10% neutral buffered formalin solution, dehydrated in alcohols and embedded in paraffin for histological and immunohistochemical evaluation. Three explants were immediately frozen in liquid nitrogen and posteriorly stored at −80 °C for the quantification of reduced glutathione (GSH), thiobarbituric acid reactive substances (TBARS), and 2,2′-azino-bis-3-ethylbenzothiazoline-6-sulphonic acid (ABTS) and ferric-reducing antioxidant power (FRAP) assays.

### 5.3. Histological and Immunohistochemical Assessment

Sections of 3 µm were stained with hematoxylin and eosin (H&E) for histopathological evaluation and scoring [12]. The microscopic changes and the frequency of the lesions were compared between the treatments as previously described [12]. The following morphological and lesional criteria were included in the score: flattening of enterocytes, villi atrophy and fusion, interstitial edema, lymphatic vessel dilation, loss of apical enterocytes, cell vacuolation, and necrotic debris. The lesional score was calculated by assessing the extent of each lesion (according to intensity or frequency observed, scored from 0 to 2; 0—diffuse, 1—moderate and 2—absent).

The measurement of the villi height was performed randomly in 10 villi per explant (100× magnification), using an image analysis system (MOTIC Image Plus Motic Instruments, Richmond, BC, Canada). Sections of the jejunum samples were subjected to periodic acid-Schiff (PAS) staining to evaluate the goblet cell density. The number of PAS stained goblet cells was counted randomly in 10 villi per explant and their respective bilateral crypts (200× magnification).

Sections from the same explants used for histopathological examination were subjected to immunohistochemical assay. Evaluations of apoptosis, cell proliferation, cell junction expression, and cox-2 expression were performed using antibodies against cleaved caspase-3 (Ccasp3) (anti-Asp 175, 1:200 dilution, Cell Signaling Technology, Beverly, MA, USA), Ki-67 (anti-7B11, 1:50 dilution, Zymed, Waltham, MA, USA), E-cadherin (anti-4A2C7, 1:50, Zymed, Waltham, MA, USA), and cox-2 (anti-CX-294, 1:100 dilution, Dako, Santa Clara, CA, USA), respectively. The protocols and positive and negative controls used were according to the manufacturer’s instructions.

A standard immunohistochemical procedure was performed. Briefly, an EDTA buffer heat-mediated (microwave oven, 750 W) antigen retrieval was used for Ki-67 and Ccasp3, and a citrate buffer for cox-2 and E-cadherin. Incubation of the sections with the primary antibody (overnight at 4 °C) was followed by incubation with the polymer secondary antibody (30 min) (Nichirei Biosciences, Tokyo, Japan), addition of the chromogen (3,3-diaminobenzidine, Invitrogen, São Paulo, SP, Brazil), and counterstaining with hematoxylin.

Immunostaining of the cytoplasm (Ccasp3) and nucleus (Ki-67) was used to assess the index of cell apoptosis (Ccasp3) and proliferation (Ki-67) in five random fields in the crypt region/explant (400× magnification). In addition, E-cadherin expression in villi enterocytes was evaluated in five fields (200× magnification). Only enterocytes showing strong, homogeneous basolateral membrane staining were considered positive. The expression of cox-2 was evaluated in the crypt region in five fields (200× magnification). Positive fields were considered when 50% or more of the cells were immunostained. The total number of fields evaluated in the three explants per treatment/animal was 15.

### 5.4. GSH Levels Measurement

GSH levels were determined spectrophotometrically by an adapted method described previously [46]. The frozen intestinal samples were homogenized using Tissue Tearor (Bjospec, São Paulo, SP, Brazil) in cold EDTA buffer (0.02 M). The homogenate was treated with 50% trichloroacetic acid (50% *w*/*v*) and centrifuged (1500× *g* for 15 min), and the supernatant was mixed with 0.4 M Tris-HCl solution (pH 8.9) and 10 mM dithiobisnitrobenzoic acid. Posteriorly, the samples were allowed to stand for 5 min before being read at 412 nm (Multiskan GO Microplate Spectrophotometer, ThermoScientific, Vantaa, Finland). A standard curve was prepared using different concentrations of GSH, in addition to the other reagents mentioned before. Results were presented as nmol GSH/mg of protein.

### 5.5. Lipid Peroxidation Measurement (TBARS)

Lipid peroxidation in the jejunum explants was assessed by determining TBARS levels, using an adapted method described by Guedes et al. [47]. For this assay, trichloroacetic acid (10%) was added to the homogenate to precipitate proteins, followed by centrifugation (1000× *g*, 3 min, 4 °C). The protein-free supernatant was separated and mixed with thiobarbituric acid (0.67%). The mixture was kept in a water bath (15 min, 100 °C). Malondialdehyde (MDA), an intermediate product of lipid peroxidation, was determined by the difference between absorbance at 535 and 572 nm using a microplate spectrophotometer reader. The results were presented as TBARS (nmol MDA/mg of protein).

### 5.6. ABTS and FRAP Assays

The ability of intestinal explants in the different treatments to resist oxidative damage was determined by the capacity to reduce 2,2′-azinobis-(3-ethylbenzothiazoline-6-sulfonate radical cation; ABTS+) (ABTS assay) and ferric-reducing antioxidant power (FRAP) assay. The frozen jejunal samples collected were homogenized in ice-cold 1.15% KCl buffer solution. Samples were centrifuged (200× *g* for 10 min at 4 °C) and the supernatants were used in both assays (ABTS and FRAP), according to Katalinic et al. [48]. The diluted ABTS solution (200 µL) was mixed with 10 µL of sample in each well. After 6 min of incubation at 25 °C, the absorbance was measured at 730 nm (Multiskan GO Microplate Spectrophotometer, ThermoScientific, Vantaa, Finland). For the FRAP assay, the supernatants (10 μL) were mixed with the prepared FRAP reagent (150 µL). The reaction mixture was incubated at 37 °C for 30 min, and the absorbance was measured at 595 nm. The results from ABTS and FRAP assays were equated using a standard Trolox curve (0.02–20 nmol). Considering these are Trolox-equivalent antioxidant capacity (TEAC) assays, results were presented as μmol Trolox-equivalent/mg of protein.

### 5.7. Statistical Analysis

The data (mean ± standard error) were analyzed using the free software Action 2.3 (Campinas, SP, Brazil). The lesional score, intestinal morphometry, goblet cells density, Ki-67, Ccasp3, E-cadherin, and cox-2 positive cells were compared by one-way analysis of variance (ANOVA), followed by Tukey’s test for multiple comparisons (*p* values ≤ 0.05). The effect of treatments on the oxidative stress response (GSH, TBARS, ABTS, and FRAP assays) was analyzed by ANOVA, followed by Duncan’s test (*p* values ≤ 0.05).

## Figures and Tables

**Table 1:** Effects of FB_1_, DON, and IP6 on oxidative stress in jejunal explants of swine.

Treatment	GSH (nmol/mg of Protein)	TBARS (ΔOD A_535_ − A_532_/mg Protein)	ABTS (nmol/Trolox Eq/mg of Protein)	FRAP (nmol Trolox Eq/mg of Protein)
Control	21.79 ± 0.49 ^a^	0.35 ± 0.04 ^bc^	60.70 ± 6.65 ^c^	60.23 ± 1.68 ^bcd^
IP6	23.24 ± 2.39 ^a^	0.21 ± 0.02 ^c^	64.00 ± 8.19 ^bc^	57.14 ± 2.77 ^bcd^
DON	8.27 ± 2.25 ^b^	0.58 ± 0.05 ^a^	40.26 ± 5.93 ^d^	51.40 ± 2.90 ^d^
DON + IP6	19.17 ± 2.37 ^a^	0.19 ± 0.02 ^c^	67.60 ± 4.19 ^abc^	66.15 ± 2.31 ^abc^
FB_1_	10.50 ± 3.66 ^b^	0.54 ± 0.03 ^a^	39.14 ± 8.66 ^d^	58.99 ± 5.25 ^bcd^
FB_1_ + IP6	21.56 ± 1.67 ^a^	0.22 ± 0.03 ^c^	81.00 ± 2.64 ^ab^	71.77 ± 1.91 ^a^
DON + FB_1_	11.67 ± 1.64 ^b^	0.43 ± 0.06 ^ab^	41.83 ± 1.09 ^d^	55.77 ± 5.35 ^cd^
DON + FB_1_ + IP6	19.26 ± 1.52 ^a^	0.23 ± 0.02 ^c^	85.75 ± 5.60 ^a^	67.71 ± 8.86 ^ab^

Mean values with their standard errors (*n*: 5 animals). Duncan’s test. Mean values with different superscript letters (column) were significantly different for each test (*p* ≤ 0.05). GSH—reduced glutathione; TBARS—thiobarbituric acid reactive substances; ABTS—2,2′-azino-bis(3-ethylbenzothiazoline-6-sulphonic acid; FRAP—ferric-reducing antioxidant power.
